# Classifying Parkinson’s Disease Based on Acoustic Measures Using Artificial Neural Networks

**DOI:** 10.3390/s19010016

**Published:** 2018-12-20

**Authors:** Lucijano Berus, Simon Klancnik, Miran Brezocnik, Mirko Ficko

**Affiliations:** Intelligent Manufactoring Laboratory, Production Engineering Institute, Faculty of Mechanical Engineering, University of Maribor, Smetanova ulica 17, Maribor 2000, Slovenia; simon.klancnik@um.si (S.K.); miran.brezocnik@um.si (M.B.); mirko.ficko@um.si (M.F.)

**Keywords:** Parkinson’s disease, feature selection, voice recognition, artificial neural networks

## Abstract

In recent years, neural networks have become very popular in all kinds of prediction problems. In this paper, multiple feed-forward artificial neural networks (ANNs) with various configurations are used in the prediction of Parkinson’s disease (PD) of tested individuals, based on extracted features from 26 different voice samples per individual. Results are validated via the leave-one-subject-out (LOSO) scheme. Few feature selection procedures based on Pearson’s correlation coefficient, Kendall’s correlation coefficient, principal component analysis, and self-organizing maps, have been used for boosting the performance of algorithms and for data reduction. The best test accuracy result has been achieved with Kendall’s correlation coefficient-based feature selection, and the most relevant voice samples are recognized. Multiple ANNs have proven to be the best classification technique for diagnosis of PD without usage of the feature selection procedure (on raw data). Finally, a neural network is fine-tuned, and a test accuracy of 86.47% was achieved.

## 1. Introduction

Parkinson’s disease (PD) is a chronic neurodegenerative disorder of the nervous system which predominantly affects motor function. It is classified as a movement disorder, with features of inability of voluntary movement (akinesis), diminished and slow movement (bradykinesis), increased muscle tonus (rigidity), and shaking movement in the resting position (Parkinson’s tremor) [1]. Some other features include diminished facial expression, problems with balance and characteristic changes of speech and voice [2,3]. People with PD can also lose sense of smell (anosmia) and have sleep disorders during the rapid eye movement sleep (REMs) phase [4]. It is estimated that PD affects around 1% of the population over 60 [5]. The cause of PD is not well understood—most of the cases have no known cause. It has been discovered that pathological changes in dopaminergic neurons and neurochemical imbalance effects are most common features of this disease. The majority of neurons producing dopamine form a black substance in the brainstem called substantia nigra [6]. This anatomical site has firm connections with other deep structures in the brain and helps to produce normal body movement. The lack of dopamine production in dopaminergic neurons of substantia nigra causes diminished range of motion, and also affects voluntary motion [7]. So far, there has been no treatment to cure PD. The disease course is variable and progresses at different rates. Symptoms of PD can be managed with various medications [8].

The diagnosis is made most commonly with neurological clinical evaluation. No laboratory parameter has been identified to detect PD, however, there is a method of nuclear imaging study that can confirm and distinguish between Parkinson’s and some other similar diseases. Nuclear imaging detects gamma radiation of various radioactive substances and can quantify signals in various parts of the brain which can confirm brain patterns in patients with PD [9]. The imaging studies have achieved high levels of recognition rates, but they are complicated and expose patients to a low dose of radiation [10,11,12]. In the majority of cases, treatment with the substance levodopa can produce good clinical response and confirms the diagnosis of PD [13].

In recent years, more research has been made in specific voice and speech patterns in people who suffer of PD [14,15]. It is estimated that more than 90% of patients have some form of speech and language disability, and this can also be one of the first signs of early PD [16]. Multiple areas of speech can be affected, such as production of spoken language (dysprosody), voice production (disphony), and articulation (dysarthria) [17,18,19,20]. There have been some characteristic patterns of atrophy and changes in vocal chords described in Parkinson’s-related hypokinetic dysarthria, which can be visualized through direct laryngoscopy [21]. The most characteristic features of Parkinson speech are silent voice, hoarseness, soft and monotonous speech, imprecise articulation, shortage of air, and tremor of the voice. Latency in response can also be observed due to slow initiation, and can be accompanied by rushes of speech. There is often decreased speech and reading rate observed during the progress of the disease [22,23]. Speech and voice can be researched through voice analysis and determination of some parameters of speech and language, such as subtle changes in voice frequencies (jitter), voice cycle-to-cycle magnitude difference (shimmer), volume (amplitude), vocal cord opening pressure etc. In terms of speech analysis, people with Parkinson’s have shorter maximum phonation time, higher jitter and shimmer, decreased pitch range and increased phonation threshold pressure [24]. 

The work that has been done on the subject of PD detection using classification algorithms is very diverse. Classification algorithms and other intelligent methods are offering experts support tools for predictions [25,26,27], although it is only possible to make accurate predictions to a certain degree [28]. There are a lot of studies done on the voice recordings, originally done at the University of Oxford by M.A. Little [29], who, in their study, sustained vowel “a” phonations recorded from 31 subjects, of whom 23 were diagnosed with PD. On those data, various classification algorithms and feature selection procedures were used [30,31,32,33,34,35,36]. The dataset used in this paper consists of different vocal terms, as far less research has been conducted on it.

The aim of this study is to evaluate the performance of neural network-based classification using different feature selections. Feature selection and dimensionality reduction is performed with self-organizing maps, principal component analysis, and on the basis of Pearson’s and Kendall’s correlation coefficient. The novelty of this study is that it offers deeper insight into how different feature selection procedures and neural network architectures influence the performance of classification to diagnose the presence of PD. 

## 2. Materials and Methods

### 2.1. Data Collection and Preprocessing

The Parkinson’s dataset used in this study is taken from the University of California at Irvine (UCI) Machine Learning Repository [37,38]. The data were collected from 20 healthy individuals (10 male, 10 female) and 20 patients with PD (14 male, 6 female) at the Department of Neurology in the Cerrahpaşa Faculty of Medicine, Istanbul University. Individual ages of healthy individuals vary between 43 and 77 (mean: 64.86, standard deviation: 8.97), and patients with PD ages vary between 45 and 83 (mean: 62.55, standard deviation 10.79). The patients are taken through a medical examination, during which they are asked by the physicians to read predefined text, including voice samples. In this context, each patient reads or says 26 voice samples containing numbers from 1 to 10, four rhymed sentences, nine words in the Turkish language, along with sustained vowels “a”, “o”, and “u” [37]. The voice samples of each patient are recorded and passed though Praat acoustic analysis software [39] to determine time frequency-based features that indicate PD with the presence of dysphonia. Table 1 shows 26 time frequency-based features extracted from each voice sample considering the previous works held on this field of study [29,30]. Recordings are made by a Trust (Dordrecht, Netherland) MC-1500 microphone with frequency range between 50 Hz and 13 kHz. The Thrust MC-1500 microphone is set to 30 dB, 96 kHz, and placed at 10 cm distance from subject [37].

**Figure 1:**
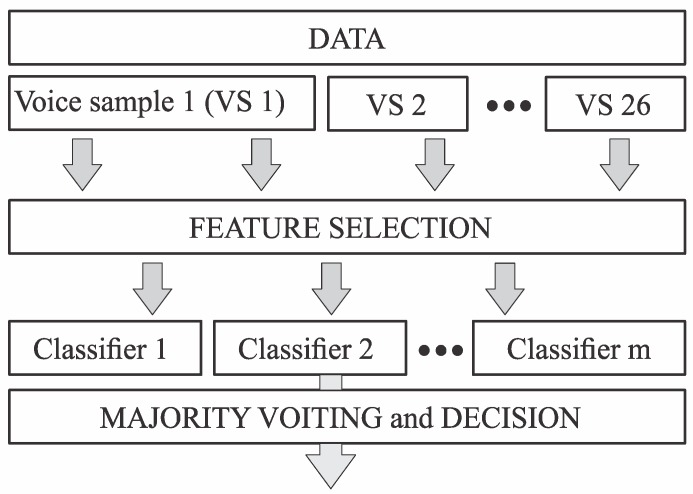
Block diagram of the proposed method.

Classification of people with PD and healthy controls is a pattern classification problem. In order to detect those patterns successfully, the data are separated into subdatasets containing tests of individuals speaking only one type of word, so-called voice samples. Then, feature selection of each voice sample is performed, with evaluating the level of influence that features have on the presence of PD. Selected features of each voice sample (m represents the number of voice samples) are then fed to a classifier. Each classifier predicts its own class label, and the final decision is made by majority voting. A block diagram of the proposed method is shown in Figure 1. Before the decision of using multiple classifiers with majority voting was made, classification with only one classifier has been performed with significantly lower recognition rates. 

### 2.2. Feature Selection Using Pearson’s and Kendall’s Correlation Coefficient

Filter-based Pearson’s and Kendall’s correlations are used for feature selection. Both methods look at how well two sets of data are correlated. Correlation simply measures the strength of the association between two variables and the direction of the relationship. Correlation shows how the variations in one set of data affect the variations in another. Pearson’s correlation is one of the most commonly used statistics to measure the relationship between related variables. It is a parametric test, meaning that it assumes the normally distributed nature of the data. It shows the linear relationship between two quantitative continuous variables. Pearson’s correlation coefficient for every feature per voice sample is calculated (this gives a matrix 26 × 26, representing correlation factors of all samples and their representative features altogether). Then, we choose to eliminate all features (per sample) that have lower than specified association. For this paper, feature selection is performed so only features with absolute values r>|0|, r>|0.25|, r>|0.30|, r>|0.35|, and r>|0.40| are considered as relevant, and other features that do not satisfy this requirement are eliminated. When high association factors are used, some voice samples are left with no representative features, therefore, whole voice samples can be omitted from the classification procedure. The features selected of a certain voice sample are then mapped linearly on the interval [−1,1] as a preprocessing step for classification.

Kendall’s correlation coefficient represents the degree of concordance between two columns of ranked data. It is a non-parametric test, as it does not rely on any assumptions on the distributions of variables. We adopt a similar procedure as in the case of Pearson’s correlation coefficient by elimination of less relevant features. Features that are considered as relevant in this study are features with $\tau_{b}>\left|0\right|,$ $\tau_{b}>\left|0.20\right|$, $\tau_{b}>\left|0.25\right|$, $\tau_{b}>\left|0.30\right|$, and $\tau_{b}>\left|0.35\right|$. The features selected of a certain voice sample are then mapped linearly on the interval [−1,1] as a preprocessing step for classification.

### 2.3. Feature Selection Using Principal Component Analysis (PCA)

PCA is a well-established statistical procedure for feature extraction and dimensionality reduction that uses an orthogonal transformation to convert a set of observations with correlated variables into a smaller set of values of linearly uncorrelated variables. It is based on the assumption that most of the information about certain classes is contained in the features with most variance. Its idea is that the p-dimensional dataset can be presented with a smaller set of n dimensions, which are presented with n leading eigenvectors of global covariance matrix [40]. In this study, the features selected (of a certain voice sample) contain all the principal components that present more than 0.1%, 0.5%, 1%, 5%, and 10% of total variance were tested.

### 2.4. Feature Selection Using Self-Organizing Map (SOM)

A self-organizing map (e.g., Kohonen network [41]) is an unsupervised learning architecture that consists of one layer, usually a two-dimensional grid of neurons. It is used as a high-dimensional data visualization tool and can be used for feature selection. The Kohonen network preserves topological properties of the dataset. The objective of the Kohonen network is to map input vectors of arbitrary dimensions onto a discrete map comprised of neurons. Unsupervised learning means that the desired output (response variable) is not presented to the network; the system is provided with group facts (patterns) and then left, to itself, to settle down to a stable state after some number of iterations [42]. Learning in the Kohonen network is performed by updating weights of a winning neuron and its neighbors. The two-dimensional topology gives us the advantage to distinguish neighborhood relationships between nodes based on distances between them.

For this study, 2 × 2, 3 × 3, 4 × 4, 5 × 5, and 6 × 6 two-dimensional SOM hexagonal grid topologies have been trained for 250 iterations using a batch unsupervised weight/bias training algorithm. The training procedure is divided into coarse and fine training. During the coarse training, the Gaussian neighborhood function radius is shrunk from 4 to 0.5 for 200 iterations. During fine training (lasting 50 iterations), the Gaussian neighborhood function radius is kept constant at 0.5. The features selected of a certain voice sample are then mapped linearly on the interval [−1,1] as a preprocessing step for classification.

### 2.5. Artificial Neural Networks (ANNs) and Classification Problems

ANNs are biologically inspired; they mimic the human brain processes and have emerged as one of the tools that can handle the classification problem. ANNs have been used to solve many problems in the Economic, Social and Engineering Sciences, as well as Health Sciences [43,44,45]. They are made of constitutive units called neurons, which are interconnected to each other with connecting links, where each link has a weight that is multiplied by the signal transmitted in the network [46]. The advantage of ANNs is that neural networks are data-driven self-adaptive methods, so that they can adjust themselves to the data without any explicit specification of functional form for the underlying model, and they can approximate any function with arbitrary accuracy [47].

An ANN consists of an input layer of nodes, one or more hidden layers, and an output layer. The input layer, in our case, consists of neurons that represent different sound parameters. The hidden layer is a collection of neurons which provide an intermediate connection between the input layer and the output layer. The hidden layer of the neural network simply maps the inputs into image space Г. The number of neurons in the output layer is determined by the number of classes. The architecture of the network is one of the most important considerations when solving problems using multilayer feed-forward neural networks. An oversimplified network architecture is less flexible [48] and might hamper the convergence of the network. On the other hand, more complex networks are much more prone to over-fitting [49,50] and, thus, poor generalization performance [51]. Besides the better generalization ability, small networks are better, because they are usually faster and cheaper to build [52]. Some books and articles offer “rules of thumb” for choosing a topology, for example, the size of the hidden layer to be somewhere between the input layer size and the output layer size, or some other rules, but such rules are total nonsense [53]. There is no way to determine a good network topology just from the number of inputs and outputs. It depends critically on the number of training cases, the amount of noise, and the complexity of the classification you are trying to learn. 

Transfer functions determine the way the signals are processed by the neurons. They are used as an integral part of the network. The transfer functions used in the majority are the sigmoidal (“*tansig*”) [54], that have non-local behavior, large activations, and they are non-zero in an infinite domain. Sigmoidal output function is smooth, so the derivatives of it exist. During the fine-tuning of a neural network algorithm, other transfer functions were also used, like “*purelin*” and “*logsig*”. Training the algorithm provides ANN with a strategy for efficient adjusting of weights belonging to a certain neuron. In our case, mostly scaled conjugate gradient backpropagation (“*trainscg*”) is used, because it is very suitable for large data processing. During the fine-tuning of our algorithm, other training algorithms were also used, like “*trainlm*” and “*trainbf*”. The strategy for preventing overfitting is the early stopping, because this method is suitable with a scaled conjugate gradient backpropagation training algorithm. 

### 2.6. Majority Voting

Since an algorithm has multiple classifiers each providing certain response if, for instance, feature selection is not used, classification with all 26 classifiers is adopted, each for a certain vocal test. Each classifier will predict the class label of its own subset; a label of “1” means the subject has PD, and “0” otherwise. The majority vote decides a class that a person belongs to. If the majority of classifiers have voted for “1”, then the subject has PD, if not, otherwise. The problem emerges when there is even number of classifiers and the result is tied. In that case, the majority voting procedure is tilted toward “1”, since it is better to examine the healthy individual further than to take no action on an individual with PD. 

### 2.7. Generalization to Unseen Data: Leave-One-Individual-Out

For the validation of our neural network model, since we do not have independent validation samples, we must build predictors using subsets of the data samples available for training and test them with the rest of the data. Using the conventional leave-one-out or bootstrapping technique [55,56,57] would result in bias in estimation. Due to the dataset structure (which consists of multiple sound recordings per person), the so-called lave-one-subject-out (LOSO) validation scheme is used. The major advantage of the LOSO is that it has far less bias, and that it provides practically unbiased prediction. The LOSO validation scheme in our neural network algorithm is established with the use of the cell array construct, so that all recordings of a particular individual are contained in separate cells. The LOSO validation scheme is then implemented by k-fold validation with 40 folds, as there are 40 individuals in the dataset.

### 2.8. Classifier Evaluation Measures

Classification is one of the most frequently encountered problems in decision-making tasks. In Machine Learning and Statistics, classification is described as the problem of identifying to which of a set of categories (subpopulations) a new observation belongs, on the basis of a training set of data containing observations (or instances) whose category membership is known. Several measures have been used in order to evaluate the effectiveness of our classification. These measures are accuracy, sensitivity, specificity, MCC, and confusion matrix. A confusion matrix [58] contains information about actual and predicted classifications done by a classification system. Table 2 shows the confusion matrix for a two-class classifier. Classification accuracy, sensitivity, specificity, and Matthews correlation coefficient (MCC) can be defined by using elements of the confusion matrix.

Accuracy is the ratio of correctly classified instances to the whole instances:

(1) $$\mathrm{accuracy}=\frac{TP+TN}{TP+FP+TN+FN},$$

where TP is the number of true positives, TN true negatives, FP false positives, and FN false negatives. Sensitivity and specificity are statistic measures of correctly classified positive and negative instances, respectively:

(2) sensitivity=TP/(TP+FN),

(3) specificity=TN/(FP+TN).

MCC is used as a measure of the quality of binary classifications. It takes into account true and false positives and negatives, and is generally regarded as a balanced measure, even if the classes are of very different size. The formulation of MCC metric is given as follows:

(4) $$\mathrm{MCC}=\frac{TP\times TN+FP\times FN}{\sqrt{\left(TP+FP\right)\left(TP+FN\right)\left(TN+FP\right)\left(TN+FN\right)}}.$$

The MCC values range between −1 and +1. The MCC coefficient is equal to +1 when a classifier makes perfect predictions, −1 when the predictions and actual values totally disagree, and 0 when the classification is no better than random prediction.

## 3. Results

Table 3 below shows the selected features of certain voice samples which have Pearson’s correlation coefficient higher than |0|, |0.25|, |0.30|, |0.35|, and |0.40|. The most relevant features which suggest the presence of PD are identified, leaving some voice samples with no related features, in the case of r>|0.25|, short sentence 1 is left with related features, therefore, the named voice sample would not be fed into the classifier later on. Some features appear multiple times as most relevant, in the case when r>|0.30|, one of the most frequent features are noise-to-harmonic ratio and jitter (ppq5) (Table 1). In order to evaluate the effectiveness of the ANN on the original feature space, no feature selection (r>|0|) has been used where all original data, meaning all voice samples and related features, are fed into 26 ANN classifiers.

Table 4 presents the selected features of certain voice samples which have Kendall’s correlation coefficient higher than |0|, |0.2|, |0.25|, |0.3|, and |0.35|. In order to evaluate the effectiveness of the ANN on the original feature space, no feature selection (t>|0|) has been used, where all original data, meaning all voice samples and related features, are fed into 26 ANN classifiers. SOM and PCA base feature selections cannot be stated in the following manner, because they transform original (time frequency-based) features in a new feature space.

Five different ANN configurations are tested, two with one hidden layer with 5 and 10 neurons (named ANN 5 and ANN 10), two with two hidden layers (ANN 5-5 and ANN 10-10), and one with three hidden layers of neurons (ANN 5-10-5). Neural networks are trained for 500 epochs, and entire LOSO cross-validation is preformed 30 times for each ANN configuration using selected features based on Pearson’s correlation coefficient, Kendall’s correlation coefficient, principal component analysis, and self-organizing maps. 

**Figure 2:**
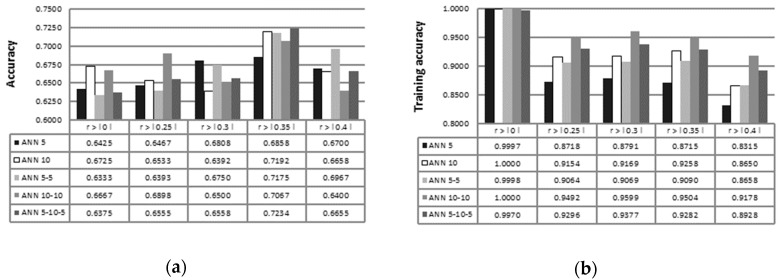
Accuracy (**a**) and training accuracy (**b**) measures of ANN 5, ANN 10, ANN 5-5, ANN 10-10, and ANN 5-10-5 configurations with Pearson’s-based feature selection.

Figure 2 presents the results of different ANN configuration for test accuracy (of the test population) and training accuracy. Accuracy of tested ANN is highly dependent on the use of feature selection; overall, the best accuracy for all tested ANN configurations is achieved with Pearson’s correlation coefficient r>|0.35|. Training accuracy decreases gradually with using higher Pearson’s correlation coefficient. On the other hand, the additional hidden layers and additional neurons increase training accuracy.

**Figure 3:**
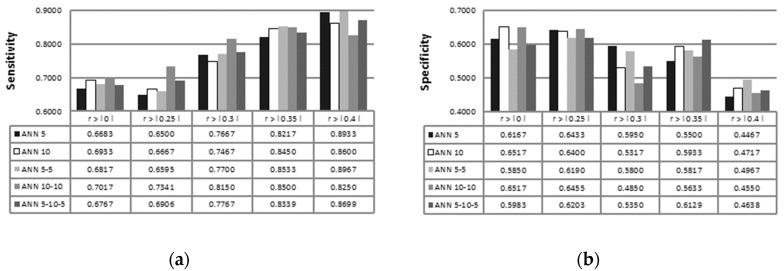
Sensitivity **(a)** and specificity **(b)** measures of ANN 5, ANN 10, ANN 5-5, ANN 10-10, and ANN 5-10-5 configurations with Pearson’s-based feature selection.

Figure 3 presents the results of different ANN configurations for sensitivity and specificity. Sensitivity is a measure of the true positive rate, and it increases gradually with increasing feature selection rate. With increasing the Pearson’s correlation coefficient, higher specificity is achieved for almost all ANN configurations. The highest sensitivity is achieved with the ANN 5-5 configuration and with r>|0.40|. Specificity defined as a measure of the true negative rate is more unstable with increasing feature selection rate. The highest specificity is achieved with the ANN 10-10 configuration and with r>|0|.

**Figure 4:**
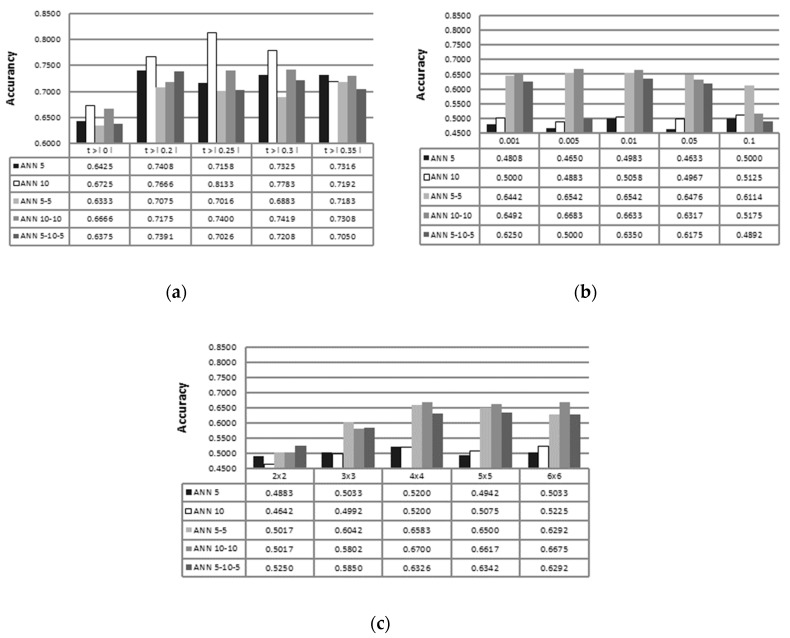
Test set accuracy of Kendall’s (**a**), PCA (**b**), and SOM based feature selection (**c**) with ANN 5, ANN 10, ANN 5-5, ANN 10-10, and ANN 5-10-5 configurations.

Test accuracies of different ANN topologies combined with feature selection techniques are stated in Figure 4. Accuracy of tested ANN is highly dependent on the use of feature selection; overall, the best accuracy (=0.8133) for all tested ANN configurations is achieved with Kendall’s correlation coefficient t>|0.25|, with ANN that consisted of one hidden layer with 10 neurons. In the case of PCA and SOM feature selection, single hidden layer ANN topologies have shown lower recognition rates, while topologies with two or three hidden layers are prone to overfitting. Best accuracy (=0.6633) of tested ANN configurations, with the use of PCA based feature selection, is achieved when features that represent more than 1% of total variance are fed to ANN 10-10 topology. 

**Figure 5:**
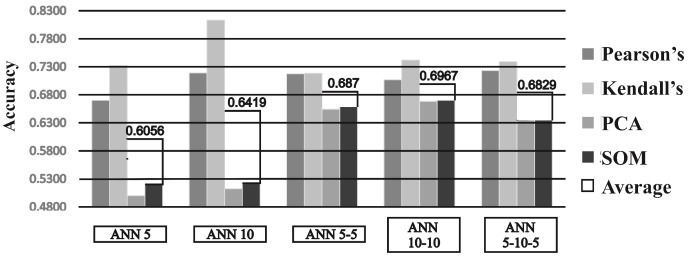
Comparison of best test set accuracies of different ANN topologies.

Different ANN topologies performances with regard to different feature selection methods are shown in Figure 5. The figure shows test set accuracies of different ANNs with best suited feature selection factors. Among all ANN topologies. Overall best average accuracy of 0.6967 was achieved with two hidden layer topology with 10 neurons in both layers (ANN 10-10).

For comparison with study [59], filter-based method named A-MCFS feature selection approach was also included. A-MCFS also use Pearson’s correlation coefficient for selection of the most relevant features; features are stated in Table 5 and, to a certain extent, satisfy Pearson’s correlation coefficient r>|0.3114|. With the use of A-MCFS, a results comparison of ANN can be made with other classifiers. Voice samples are fed into the classification algorithm. Neural networks are fine-tuned using different combinations of training algorithms, transfer functions, and topologies. Fine-tuning is performed with the desire to increase the test accuracy, while obtaining high levels of sensitivity and specificity.

The best results achieved in this study are stated in Table 6. The Table also offers a comparison of different results with other studies. The performance of ANN using feature selection scheme has been enhanced in the case of using Pearson’s correlation coefficient and Kendall’s correlation coefficient, while PCA-ANN and SOM-ANN structures have shown lower recognition rates. In the case of PCA-ANN and SOM-ANN, feature selection is performed by transformation of input patterns to a lower dimensional space. Transformation takes place without taking into account the response variable. It should be stated that transformation creates new features that, to some extent, resemble properties of primal time frequency-based features in the newly developed lower dimensional feature space. Training accuracies for A-MCFS (fine-tuned), Kendall’s ANN, PCA-ANN, and SOM-ANN (listed in Table 6) have been 89.43%, 87.51%, 100%, and 100% respectively. Using Kendall’s correlation coefficient for feature selection, 81.33% accuracy is achieved with $\tau_{b}>\left|0.25\right|$. The best results for PCA-ANN were achieved by taking into account all principal components that present more than 1% of total variance of the dataset (that resulted in feeding 26 classifiers with, on average, 17 first principal components extracted from voice samples). The highest test accuracies of SOM-ANN are achieved with 4×4 hexagonal self-organizing map topology. In the case of SOM-based feature selection, the ANN training rate is quite dependent on the number of hidden layers of ANN. Using SOM-based feature selection with only one ANN hidden layer topology, low training accuracies have been achieved, varying from 50.95% to 57.11%.

## 4. Discussion

In this work, multiple ANNs with feature selection based on Pearson’s correlation coefficient, Kendall’s correlation coefficient, PCA, and SOM have been developed for addressing the PD diagnosis problem. The multiple ANN algorithms are used to classify the individuals into classes. Each subject is classified into the class “healthy” or “PD” based on the majority voting procedure. In Machine Learning, one of the problems is identifying a representative set of features from which to construct a classification model for a particular task. With using feature selection, the procedure size of the problem is reduced by reducing the dimensionality of the data, and improvement of ANN performance can then be achieved by removing the noisy or irrelevant features and preventing the overfitting to noisy data. Using more hidden layers and adding more neurons to existing layers has been proven to alter the result, meaning that appropriate ANN response is dependent on ANN architecture. The same thing can be concluded for usage of certain types of feature selection procedure. With regard to statistical significance of results, no claims can be made because extensive statistical tests were omitted.

It was observed that multiple ANNs achieved the highest accuracy among classifiers of 67.25% via LOSO cross-validation using no feature selection. Discussed accuracy was achieved with one dimensional ANN 10 architecture, while achieving training accuracy of 100%. Multiple ANNs have achieved the second highest accuracy among classifiers of 86.47% via LOSO cross-validation with using the A-MCFS filter-based feature selection method and fine-tuning the procedure. During this procedure, different configurations were used for each ANN classifier (among 15 ANN classifiers), meaning different training algorithms, learning rates, number of learning epochs and architectures. The highest ANN test accuracy of 81.33% has been achieved with one dimensional ANN 10 topology and Kendall’s correlation coefficient-based feature selection. Best multiple ANN accuracies, with the use of Pearson’s correlation coefficient-, PCA-, and SOM-based feature selections, were all achieved with two hidden layer neural network architecture ANN 10-10, which has also proven to be the most suited ANN topology for addressing the PD diagnosis problem.

PCA-ANN and SOM-ANN achieved similar recognition rates, and were surpassed by correlation coefficient-based feature selection procedures. It was shown that one-layer ANN topologies, with PCA and SOM feature selections, could not adequately model the PD problem, consequently achieving low test and training accuracies. Feature selection with Kendall’s and Pearson’s correlation coefficient enhanced multiple ANNs accuracies. Based on fact that multiple ANNs with Kendall’s correlation coefficient surpassed accuracies of multiple ANNs with Pearson’s correlation coefficient, we can conclude that the data have no normally distributed nature. Some of the voice samples used by other authors for determining presence of PD, such as vowel “a”, have been shown to carry little information. We can state, based on Pearson’s and Kendall’s correlation coefficients, that voice samples of “number 4” and “short sentence 4” have been recognized to carry the most information about PD. This may indicate that more information about the presence of PD is imbedded in voice samples consisting of more diverse sounds compared to simple sounds like sustained vowels pronunciations. The excellent performance obtained on the PD dataset has proven that the proposed system can distinguish well enough between patients with PD and healthy individuals. It can be concluded safely that developed ANNs can, to some extent, assist physicians to make accurate diagnostic decisions.

As an extension of this study, we suggest a research direction for future work: it is possible to improve the ANNs’ performance by using other feature selection procedures and by additional work on fine-tuning. Future work should also be oriented into collecting several vocal tests in other languages and performing the classification on those datasets. A described approach for detection of PD is, in this stage, clearly experimental and cannot, by its own, be used for clinical diagnosis.

## Figures and Tables

**Table 1:** Extracted time frequency-based features from individual voice samples [37].

Feature Number	Feature	Mean	Stand. Deviation
1	Jitter (local)	2.67952	1.76505
2	Jitter (local, absolute)	0.00017	0.00011
3	Jitter (rap)	1.24705	0.97946
4	Jitter (ppq5)	1.34832	1.13874
5	Jitter (ddp)	3.74116	2.93844
6	Number of pulses	12.91839	5.45220
7	Number of periods	1.19489	0.42007
8	Mean period	5.69960	3.01518
9	Standard dev. of period	7.98355	4.84089
10	Shimmer (local)	12.21535	6.01626
11	Shimmer (local, dB)	17.09844	9.04554
12	Shimmer (apq3)	0.84601	0.08571
13	Shimmer (apq5)	0.23138	0.15128
14	Shimmer (apq11)	9.99954	4.29130
15	Shimmer (dda)	163.3683	56.02168
16	Fraction of locally unvoiced frames	168.7276	55.96991
17	Number of voice breaks	27.54763	36.67262
18	Degree of voice breaks	134.5381	47.05806
19	Median pitch	234.8760	121.5412
20	Mean pitch	109.7442	150.0277
21	Standard deviation	105.9692	149.4171
22	Minimum pitch	0.00655	0.00188
23	Maximum pitch	0.00084	0.00072
24	Autocorrelation	27.68286	20.97529
25	Noise-to-harmonic	1.13462	1.16148
26	Harmonic-to-noise	12.37001	15.16192

**Table 2:** Confusion matrix representation.

	Predicted	
**Actual**	Positive	Negative
Positive	TP	FN
Negative	FP	TN

**Table 3:** Selected time frequency-based features using selected Pearson’s correlation factors in the case of testing multiple artificial neural networks (ANNs) on subject no. 1, and training of ANNs is performed on the other 39 subjects.

ID	Voice Sample	Related Features (r >|0|)	Related Features (r >|0.25|)	Related Features (r >|0.30|)	Related Features (r >|0.35|)	Related Features (r >|0.4|)
1	Vowel “a”	All	24	None	None	None
2	Vowel “o”	All	19, 24	24, 19	None	None
3	Vowel “u”	All	13, 21	None	None	None
4	Number 1	All	1, 2, 3, 4, 5, 24	1, 2, 3, 4, 5, 24	1, 2, 4	1, 4
5	Number 2	All	1, 2, 8, 9, 10, 11	2, 8, 9, 10, 11	10	None
6	Number 3	All	12, 13, 14, 17, 19, 23, 25, 26	17, 19, 23, 25, 26	17, 19, 23, 25, 26	17, 25
7	Number 4	All	1, 2, 3, 4, 5, 10, 20, 21	1, 2, 3, 4, 5, 10	1, 2, 3, 4, 5	1, 2, 3, 4, 5
8	Number 5	All	24	24	24	None
9	Number 6	All	10, 23, 26	None	None	None
10	Number 7	All	17, 19, 24, 26	None	None	None
11	Number 8	All	9, 10	9	None	None
12	Number 9	All	26	26	None	None
13	Number 10	All	1, 2, 3, 5, 8, 9, 11, 23	None	None	None
14	Short sentence 1	All	None	None	None	None
15	Short sentence 2	All	3, 4, 5, 24, 25, 26	25, 26	25	25
16	Short sentence 3	All	3, 4, 5, 10, 25, 26	4, 10, 25, 26	10, 26	26
17	Short sentence 4	All	1, 2, 3, 4, 5, 10, 24, 25, 26	1, 2, 3, 4, 5, 10, 26	1, 2, 3, 4, 5, 10, 26	3, 4, 5, 10
18	Word 1	All	1, 2, 4, 7	1, 2	None	None
19	Word 2	All	10	None	None	None
20	Word 3	All	17, 19, 23, 25	17, 19, 23, 25	17, 19	17, 19
21	Word 4	All	3, 5	None	None	None
22	Word 5	All	26	26	None	None
23	Word 6	All	2, 10	None	None	None
24	Word 7	All	17	None	None	None
25	Word 8	All	1, 2, 3, 4, 5, 10, 17, 19, 23, 24, 25	1, 2, 3, 5, 17, 19, 23, 25	4, 17, 19	17, 19
26	Word 9	All	2, 24	24	None	None
Number of classifiers		26	25	16	10	8

**Table 4:** Selected time frequency-based features using selected Kendall’s correlation factors in the case of testing multiple ANNs on subject no. 1, and training of ANNs is performed on the other 39 subjects.

ID	Voice Sample	Related Features (t >|0|)	Related Features (t >|0.2|)	Related Features (t >|0.25|)	Related Features (t >|0.3|)	Related Features (t >|0.35|)
1	Vowel “a”	All	6, 7, 9, 10, 14	10	None	None
2	Vowel “o”	All	17, 24	24	24	24
3	Vowel “u”	All	24	24	None	None
4	Number 1	All	1, 2, 3, 4, 5, 6, 7, 9,10, 24	1, 2, 3, 4, 5, 6, 24	1, 2, 4, 24	None
5	Number 2	All	1, 2, 3, 4, 5, 6, 8, 9, 10, 11	1, 8, 9, 10, 11	9	None
6	Number 3	All	12, 13, 14, 17, 19, 23, 24, 25, 26	12, 13, 17, 19, 23, 25, 26	17, 23, 25, 26	17,25,26
7	Number 4	All	1, 2, 3, 4, 5, 10, 20, 21	1, 2, 3, 4, 5, 10,	1, 2, 3, 4, 5,	1,2,3,4,5
8	Number 5	All	24	24	24	None
9	Number 6	All	10, 24, 26	10, 26	None	None
10	Number 7	All	1, 3, 4, 5, 8, 11, 24	4, 5	4	None
11	Number 8	All	9	9	9	None
12	Number 9	All	2, 3, 4, 5, 21, 26	4, 26	4	None
13	Number 10	All	1, 3, 5, 20, 23	23	None	None
14	Short sentence 1	All	25, 26	None	None	None
15	Short sentence 2	All	3, 4, 5, 8, 10, 11, 17, 25, 26	24, 25, 26	25	25
16	Short sentence 3	All	1, 2, 3, 4, 5, 10, 17, 24, 25, 26	10, 26	26	None
17	Short sentence 4	All	1, 2, 3, 4, 5, 10	1, 2, 3, 4, 5, 10	1, 3, 4, 5, 10, 25, 26	3,5
18	Word 1	All	1, 2, 3, 4, 5, 7	1, 2, 4, 7	1, 4	None
19	Word 2	All	None	None	None	None
20	Word 3	All	17, 19, 23, 25	17, 19, 25	17, 25	17
21	Word 4	All	3, 5	None	None	None
22	Word 5	All	17, 19, 26	None	None	None
23	Word 6	All	10, 17	10	None	None
24	Word 7	All	3, 5, 23	None	None	None
25	Word 8	All	1, 2, 3, 4, 5, 10, 14, 17, 19, 23, 25	2, 17, 19, 25	17, 19	17
26	Word 9	All	2, 3 4, 5, 24	24	None	None
Number of classifiers		26	25	21	15	7

**Table 5:** Selected features using a medium correlation factor.

ID	Voice Sample	Related Features
1	Vowel “a”	None
2	Vowel “o”	24
3	Vowel “u”	None
4	Number 1	1, 2, 3, 4, 5, 24
5	Number 2	2, 9, 10
6	Number 3	17, 19, 23, 25, 26
7	Number 4	1, 2, 3, 4, 5, 10
8	Number 5	24
9	Number 6	None
10	Number 7	None
11	Number 8	9
12	Number 9	26
13	Number 10	None
14	Short sentence 1	None
15	Short sentence 2	25, 26
16	Short sentence 3	4, 10, 25, 26
17	Short sentence 4	1, 2, 3, 4, 5, 10, 26
18	Word 1	2
19	Word 2	None
20	Word 3	17, 19, 23, 25
21	Word 4	None
22	Word 5	None
23	Word 6	None
24	Word 7	None
25	Word 8	1, 2, 3, 5, 6, 17, 19, 23, 25
26	Word 9	24
Number of classifiers		15

**Table 6:** Comparison of different classifiers performance on PD dataset.

Classifier	Feature Selection	Accuracy (%)	Sensitivity (%)	Specificity (%)	MCC
k-NN (k = 1)	/ [37]	53.37	49.62	57.12	0.0007
	A-MCFS [59]	70.00	80.00	60.00	0.4082
k-NN (k = 3)	/ [37]	54.04	53.27	54.81	0.0008
	A-MCFS [59]	67.50	75.00	60.00	0.3540
k-NN (k = 5)	/ [37]	54.42	53.65	55.19	0.0009
	A-MCFS [59]	72.50	70.00	75.00	0.4506
k-NN (k = 7)	/ [37]	53.94	54.04	53.85	0.0008
	A-MCFS [59]	77.50	80.00	75.00	0.5507
SVM (linear kernel)	/ [59]	52.50	52.50	52.50	0.0006
	A-MCFS [59]	85.00	85.00	85.00	0.6000
SVM (RBF kernel)	/ [59]	55.00	60.00	50.00	0.1005
	A-MCFS [59]	87.50	90.00	85.00	0.7509
ANN 10	/	67.25±4.52	69.33±6.66	65.17±5.65	0.3467±0.090
ANN 5-10-5	Pearson’s	72.34±4.54	83.39±7.14	61.29±5.86	0.4610±0.096
ANN 10	Kendall’s	81.33±4.58	86.33±6.56	76.33±5.40	0.6318±0.093
ANN 10-10	PCA	66.33±4.99	67.83±8.48	64.83±6.23	0.3288±0.100
ANN 10-10	SOM	67.00±4.28	69.50±5.78	64.50±6.61	0.3417±0.085
ANN (fine-tuned)	A-MCFS	86.47±3.27	88.91±4.79	84.02±5.10	0.7321±0.064
